# Marginal Bone Level Evaluation of Fixed Partial Dental Prostheses Using Preformed Stock versus CAD/CAM Customized Abutments

**DOI:** 10.3390/jpm12071051

**Published:** 2022-06-27

**Authors:** Hui-Ting Lin, Jerry Chin-Yi Lin, Eisner Salamanca, Odontuya Dorj, Yu-Hwa Pan, Yi-Fan Wu, Yung-Szu Hsu, Chih-Yuan Fang, Wei-Jen Chang

**Affiliations:** 1 School of Dentistry, College of Oral Medicine, Taipei Medical University, Taipei 110, Taiwan; m204109007@tmu.edu.tw (H.-T.L.); drjerrylin@gmail.com (J.C.-Y.L.); eisnergab@tmu.edu.tw (E.S.); dorj.odontuya@gmail.com (O.D.); shalom.dc@msa.hinet.net (Y.-H.P.); yfwu@tmu.edu.tw (Y.-F.W.); nm8346@yahoo.com.tw (Y.-S.H.); 2 Department of Oral Medicine, Infection and Immunity, Harvard School of Dental Medicine, Boston, MA 01238, USA; 3 Department of Dental technology and Dental Hygiene, School of Dentistry, Mongolian National University of Medical Sciences, Ulaanbaatar 14210, Mongolia; 4 Department of Dentistry, Chang Gung Memorial Hospital, Taipei 105, Taiwan; 5 Graduate Institute of Dental & Craniofacial Science, Chang Gung University, Taoyuan 333, Taiwan; 6 School of Dentistry, College of Medicine, China Medical University, Taichung 404, Taiwan; 7 Dental Department, Taipei Medical University, Shuang Ho Hospital, New Taipei City 235, Taiwan; 8 Department of Dentistry, Taipei Municipal Wan Fang Hospital, Taipei 110, Taiwan

**Keywords:** customized abutments, preformed abutments, functional loading, marginal bone level

## Abstract

Background: The maintenance of marginal bone levels around dental implants is an important criterion for evaluating the success of implants. Although computer-aided design/computer-aided manufacturing (CAD/CAM) customized abutments (CAs) provide more flexible solutions, compared with the original preformed stock abutments (PAs), there are dimensional tolerances and underlying drawbacks in the production of CAD/CAM CAs, which may change the tightness and seamless connection between fixtures and abutments set by the manufacturer and then affect the long-term stability of the abutments. This study aimed to examine the change in both mesial and distal bone levels using digital periapical radiographs to evaluate the difference between CAD/CAM CAs and original PAs.Material and methods: Radiographs were taken before delivery; after functional loading for 1 month; and after 3, 6, and 12 months; and the vertical marginal bone levels (vMBLs) of both the mesial and distal surrounding implant bones were measured. All data are presented as means ± standard errors and were analyzed using Student’s *t*-test. A *p*-value < 0.05 was judged to represent a significant difference. Results: A total of 57 implants in 50 patients were divided into 22 CAD/CAM CAs and 35 original stock abutments. The PAs appeared to have a more stable bone level. By contrast, the amount of bone level change in the CAs was higher than that in the PAs. The change in the vMBL of the CAs was significantly more than that of the PAs after functional loading for 1 month (*p* = 0.006), 3 months (*p* = 0.013), 6 months (*p* = 0.014), and 12 months (*p* = 0.002). In contrast, the distal marginal bone level was lower than the mesial marginal bone level in any period. Nevertheless, the bone levels of the CAs and PAs in any period were comparable with no significant difference. Conclusions: Significant differences were found between the mesial and distal bone levels in the PAs. The CAD/CAM CAs showed a significantly greater bone level change than the original stock abutments after functional loading.

## 1. Introduction

Dental implants can provide many dental clinical treatment options and their applications include single-tooth replacement and full-mouth rehabilitation. Modern dental implants can be traced back to 1965 when Professor Brånemark announced that he had performed the first dental implantation and followed this for 15 years, which laid the foundation for implant success [1]. The implant material was commercially pure titanium, and its first application in dentistry was in an edentulous ridge, providing an effective solution for restoring the esthetics and occlusal functions of missing teeth. Nowadays, implant therapy is generally recognized as a reliable and predictable tool for dental rehabilitation. However, it requires multiple factors for maintaining long-term success and esthetics.

Albrektsson and Zarb proposed the criteria for implant success to identify clinical evidence of osseointegration and implant survival [2]. Implant success was judged in terms of implant stability, the absence of peri-implant radiolucency on radiographs, <2 mm bone loss in the first year and <0.2 mm annually thereafter, and infection-free soft tissues around the dental implants. After decades, these criteria are still used. To understand osseointegration for better implant selection, studies on the surface characteristics and osseointegration of dental implants were published over the years [3,4,5]. More specifically, Marco Degidi published several articles on XiVE^®^ implants with long-term clinical follow-ups. Regarding whether immediate functional loading can have effects, all implants successfully achieved osseointegration, both clinically and radiographically [6], showing the safety and reliability of XiVE^®^ implants with a few complications [7,8,9]. Giuliao et al. published data regarding long-term follow-ups for up to 15 years after platform-matching implants subcrestally with a survival rate of 97.3% and demonstrated that no implant was lost because of peri-implantitis [10]. Nevertheless, several studies mentioned that submerged bone-level implants had higher mean marginal bone resorption and less bone-to-implant contact than non-submerged tissue-level implants [11]. Supracrestally placed implants generate less bone loss than crestally placed implants, with significant differences in both platform-switched and platform-matched implants [12,13]. The bone-level change was also associated with an implant prosthetic connection, appeared to be more effective for better vertical and horizontal stability of peri-implant alveolar bones with platform-switched implants [14], demonstrated significantly less bone loss in comparison to non-platform-switched implants [15], and increased the long-term success of dental implants [16].

The presence of a bacterial biofilm is also an influencing factor for the implant marginal bone level (MBL) [17]. Unfortunately, a biofilm occurs on all implant surfaces and is associated with peri-implantitis. Two-piece implants cannot avoid the presence of a micro-gap between the fixture and the abutment. A recent systematic review concluded that the position of a micro-gap may influence crestal bone-level changes [18]. Related studies mentioned that inaccurately sealed connections may be wobble and cause the pumping effect. Compared with the use of original parts, the use of compatible abutments may significantly increase the micromovements between the abutments, and the inner part of the implant may increase the stress on the MBL. This resulting pumping effect can transport microorganisms. Clinically, when associated with leakage, these micromovements lead to bone loss around the neck of the implant and later to peri-implantitis [19].

In recent years, with the evolution of computer-aided design/computer-aided manufacturing (CAD/CAM), customized abutments (CAs) have been increasingly used since they provide solutions to many implant-related problems. Surgeons and restorative dentists maintain the ideal emergence profile for optimal final restorative contours [20]. A previous related study mentioned that the adoption of CAD/CAM titanium abutments has many advantages when compared with the use of traditionally casted custom abutments. However, long-term in vivo and in vitro studies are necessary to verify the physical and biological characteristics and long-term performance [21]. CAD/CAM CAs can provide more flexible solutions and are widely used, but despite their merits, they have some complications, for instance, abutment loosening [22,23] and lower marginal accuracy [24]. Whether these complications will affect the long-term success of dental implants is yet to be studied. Therefore, retrospective studies of radiographic film outcomes that determine criteria to evaluate the reliability of CAs in the future are necessary.

Implant stability and the maintenance of a stable crestal bone level are preconditions for the long-term successful function of dental implants, and continuous crestal bone loss represents a threat to the longevity of implant-supported prosthetic reconstructions. Regardless of the cause of continuous crestal bone loss, radiographs are of scientifically crucial importance for evaluating dental implants. The periapical radiographic technique is currently the preferred method for evaluating implant health based on bone loss, and digital radiographs allow for easy standardization of the image contrast. After taking baseline radiographs, follow-up radiographs should be taken annually thereafter [25]. The measurement method involving measuring the distance of the mesial and distal MBLs from the fixture platform to the alveolar ridge was commonly used in previous studies [26].

While previous research summed up relevant data, data were limited to preformed stock abutments (PAs), while data was lacking for CAs. Therefore, obtaining clinical retrospective data will help in evaluating the use of CAs in the future. In this study, we aimed to investigate the difference between CAD/CAM CAs and original stock abutment interference in mesial and distal bone-level changes using digital periapical radiographs. We also tested the hypothesis that CAD/CAM CAs would present more bone loss in this study.

## 2. Material and Methods

In this retrospective study, radiographs were obtained from the Taipei Medical University Shuang Ho Hospital, New Taipei, Taiwan. The study protocol was registered and approved by the Taipei Medical University Joint Institutional Review Board (registration no. N202103105).

### 2.1. Inclusion and Exclusion Criteria

We followed the instituted criteria of the American Academy of Periodontology and enrolled adults aged > 18 years who received dental implant treatment with cement-type prosthetics.

The inclusion criteria were as follows: no systemic conditions, such as diabetes; good oral hygiene; non-smokers or smoking < 10 cigarettes per day; sufficient bone tissue; keratinized tissue level > 2 mm at the time of the implant surgery; and the absence of inflammation surrounding the operation site. The exclusion criteria were as follows: previous and current radiotherapy in the head area < 12 months before the study, long-term oral medication or intravenous use of amino-bisphosphonates, pregnancy or breastfeeding, smoking > 10 cigarettes a day, chewing of betel nuts or tobacco, alcoholism, bruxism and clenching, periodontal disease, excessive bone augmentation before implant surgery, implant failure, and an unwillingness to return for follow-up oral radiographs.

### 2.2. Surgical Procedure and Prosthetics Delivery of Implants

A complete dental implant treatment included a fixture placement, soft tissue shaping, an impression being taken, prosthetic fabrication, and the final delivery. Since the time required for osseointegration varied from 8 weeks to 6 months because of the different physical conditions of the patients, the study involving the period from impression taking to functional loading. All fixtures were XiVE implants (DENTSPLY SIRONA Implants, Hanau, Germany). After complete osseointegration, the second stage of the transgingival surgery was performed, and after 2 weeks of soft tissue shaping, the impression was taken. The prosthetics were delivered 2 weeks after the impression was taken. Implant surgery and prosthetic delivery were performed by the same experienced dentist, and the abutments were screwed with a torque wrench at 24 N/cm in accordance with the manufacturer’s instruction standard. All prosthetics were cement-type porcelain-fused-to-metal restorations.

These implants were mainly divided into two groups according to the different abutments used in the patients: the CAD/CAM CA group received CA implants and the PA group received PA implants. The raw material of the CAs was titanium, which was produced by ARCH Dental Laboratory Co., Ltd. in Taipei, Taiwan. For the PAs, the XiVE^®^ implant system EstheticBase was used, and the raw material was grade 2 commercially pure titanium. The manufacturer was DENTSPLY SIRONA Implants, which is located in Hanau, Germany.

### 2.3. Acquisition of Radiographs

**Figure 1:**
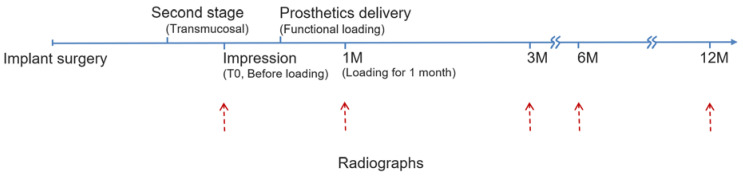
Flow chart.

Follow-up radiographic images, which were essential for the minimization of the changes in the surrounding bone level, were taken. The time of collecting radiographs when the impression was taken before functional loading was referred to as T0; the prosthetics were regularly delivered after 2 weeks. In order to fix the time of collecting radiographs, it was uniformly set at 1.5 months after the impression was taken as the reference point of functional loading for one month, and so on for the further 3, 6, and 12 months. (Figure 1). In this study, measurements were mainly made of the bone level demonstrated on the radiographs; digital radiographs were taken with periapical film (PA) in a parallel method by experienced radiologists.

Since we had records of the implant size of each patient, we used the diameter and length of the implants as a scale to correct the image ratio. After obtaining the digital radiographs, the EZ-Dental Professional Image Software (Asahi Co., Ltd., Kyoto, Japan) was used for calibration by inputting the actual length of the fixtures, and then the mesial and distal peri-implant bone levels were measured.

### 2.4. Benchmark for Measuring MBLs

The measurement method was based on a previous study [26] in which both the mesial and distal MBLs were measured from the fixture platform to the alveolar ridge.

**Figure 2:**
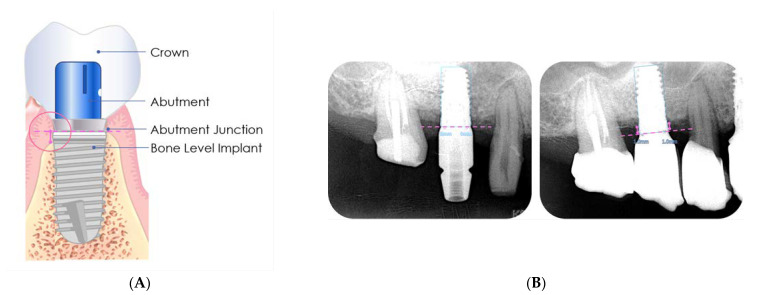
Baseline measurement (**A**). Radiographic measurement of the mesial and distal marginal bone levels (**B**).

The baseline of the reference point was located at the junction between the fixture and the abutment. The vertical height distance from the fixture–abutment junction to the margin of the crestal bone was measured (Figure 2) and recorded in mm.

The amount of change in the MBL was measured by comparing the mean MBLs before prosthetics delivery and after different follow-up periods. The average change in the mesiodistal bone levels was calculated for each dental implant.

### 2.5. Statistical Analysis

After the data were obtained, a series of comparisons were made, where the statistical analysis was conducted using Excel (Microsoft Office 2016, Microsoft Corporation, WA, USA). All data are presented as mean ± standard error. Data were analyzed using Student’s *t*-test, and a probability value (*p*-value) < 0.05 was judged to represent a significant difference.

## 3. Results

The demographics of the patients and relevant implant information are shown in Table 1. In this study, 33 patients received PAs, of which 16 were men and 17 were women. Moreover, 17 patients received CAD/CAM CAs, of which 11 were men and 6 women. In total, the study included 50 patients (Table 1). The 57 total implants were divided into the PA group with 35 implants (17 in the maxilla and 18 in the mandible) and the CAD/CAM CA group with 22 implants, which were located in the upper jaw region. After filtering, all implants were in the posterior area, where the implant diameters were 3.8 mm in 28 implants and 4.5 mm in 29 implants, and the lengths of all implants were 9.5 mm and 11 mm, respectively (Table 2). All dental implants survived, and during this period, no implants failed because of peri-implantitis or other complications.

### 3.1. Different MBLs between the Mesial and Distal Bones

**Figure 3:**
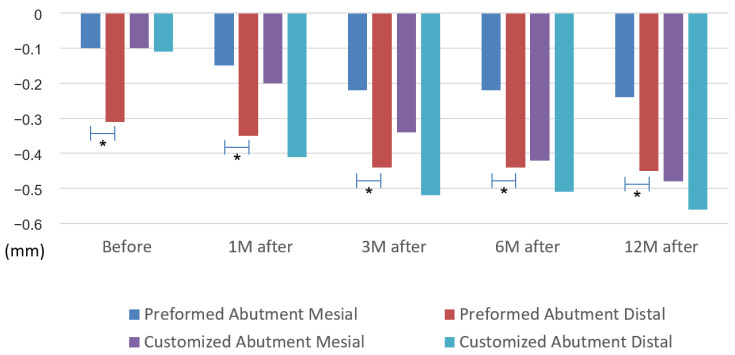
Measurement data of mesial and distal bone levels in each period. (* *p* < 0.05, by two-tailed *t*-test.).

Bone resorption occurred in each period when comparing the mesial and distal MBLs in the PA and CA groups. In the PA group, the mesial MBL before the functional loading was below the baseline with 0.10 ± 0.03 mm, and the distal MBL was 0.31 ± 0.07 mm. At 1 month after the functional loading, the MBLs were 0.15 ± 0.05 mm and 0.35 ± 0.07 mm, and at 3 and 6 months after the functional loading, the values were 0.22 ± 0.06 mm and 0.44 ± 0.08 mm, respectively. Finally, at 12 months after the functional loading, the mesial and distal MBLs were 0.24 ± 0.06 mm and 0.45 ± 0.08 mm, respectively. Moreover, the *p*-values were 0.012 before the functional loading, *p* = 0.029 at 1 month after the functional loading, *p* = 0.027 at 3 months, *p* = 0.024 at 6 months, and *p* = 0.041 at 12 months. A significant difference was found between the mesial and distal MBLs in the PA group, but no significant difference was found in the CA group. However, a slight difference was noted, as shown in Table 3 and Figure 3.

### 3.2. Comparable Bone Levels between CAs and PAs

**Figure 4:**
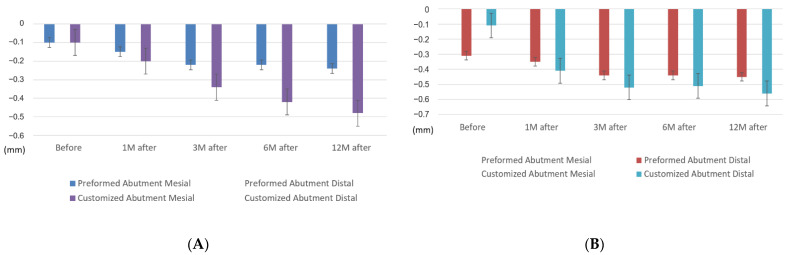
Comparison of the mesial marginal bone levels between the CAs and PAs (**A**) and the distal marginal bone levels (**B**). CA, customized abutment; PA, preformed stock abutment.

The mesial and distal bone levels both decreased in the PAs and CAs. The mesial MBL before the functional loading in the CA group was 0.10 ± 0.07 mm, which was comparable with that of the PA group with 0.10 ± 0.03 mm. At 3 months after the functional loading, the CA group was likely to have a lower bone level (0.34 ± 0.10 mm) than the PA group (0.22 ± 0.06 mm). Specifically, the mesial MBL of the CA group 12 months after the functional loading (0.48 ± 0.12 mm) was much lower than that of the PA group (0.24 ± 0.06 mm). The distal MBL was approximately 0.44 mm in the PA group and 0.52 mm in the CA group during the first 6 months after the functional loading, and 0.45 ± 0.08 mm in the PA group and 0.56 ± 0.12 mm in the CA group at 12 months after the functional loading. However, no significant difference was found between the two groups (Table 4, Figure 4).

### 3.3. Moderate Marginal Bone Loss in PAs

Table 5 presents the mesial and distal MBLs in each period in the PA group. As shown, the mesial MBL was 0.10 ± 0.03 mm before the functional loading, which increased to 0.24 ± 0.06 mm in the 12 months after the functional loading. Despite the slight bone resorption, no significant differences were found (*p* > 0.05). In contrast, the distal MBL was 0.31 ± 0.07 mm before the functional loading and 0.45 ± 0.06 mm at 12 months after the functional loading. When comparing each period, no significant difference was found between the mesial and distal MBLs in the PA group. Although marginal loss occurred, no rapid or massive loss occurred in any period (Table 5).

### 3.4. Significantly Greater Marginal Bone Loss in CAs

**Figure 5:**
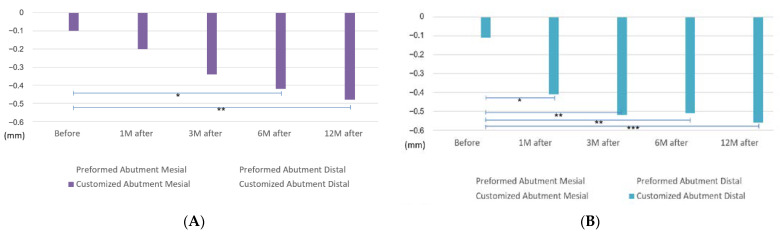
Comparison of the mesial (**A**) and distal (**B**) marginal bone levels in the CAs. CA, customized abutment. (* *p* < 0.05, **p < 0.01, ***p < 0.001, by two-tailed *t*-test.)

The mesial and distal MBLs of the CAs were reduced. In the beginning, before the functional loading, the mesial MBL was 0.10 ± 0.07 mm. At 1 month after the functional loading, it was 0.20 ± 0.09 mm, which appeared to decrease more than the initial MBL. At 6 months after the functional loading, the mesial MBL was 0.42 ± 0.10 mm (*p* = 0.011), and it was 0.48 ± 0.12 mm (*p* = 0.006) at 12 months after the functional loading, where both values were significantly lower than the initial MBL. Regarding the distal MBL, it was 0.11 ± 0.05 mm before the functional loading but dropped significantly after 1 month to 0.41 ± 0.11 mm (*p* = 0.018). Twelve months after the functional loading, the value was 0.56 ± 0.12 mm (*p* < 0.001). A significant decline in the distal MBL was noted in each period after the functional loading (Table 6, Figure 5).

### 3.5. Change in MBLs

The changes in the mesial and distal MBLs were comparable in the PA group, but the distal MBL was greater than the mesial MBL in the CA group. When comparing the changes between the mesial MBL and the distal MBL, we found that the reduction trends were comparable in the PA group; the changes were 0.05 ± 0.03 mm and 0.04 ± 0.03 mm (*p* = 0.792) in the mesial and distal MBLs, respectively, at 1 month after the functional loading, and approximately 0.12 mm in the mesial MBL and approximately 0.13 mm in the distal MBL at 3–6 months after the functional loading. At 12 months after the functional loading, the MBL change was approximately 0.14 mm for both the mesial and distal MBLs. The distal MBL changed slightly more than the mesial MBL in the CA group. The changes in the CA group were 0.10 ± 0.07 mm and 0.30 ± 0.11 mm in the mesial and distal MBLs, respectively (*p* = 0.119), at 1 month after the functional loading and 0.41 ± 0.13 mm and 0.52 ± 0.14 mm in the mesial and distal MBLs, respectively (*p* = 0.575). The change in the distal MBL appeared to be higher than that in the mesial MBL, even though no significant difference was found (Table 7).

When comparing the amount of MBL change between the PA group and the CA group, the mesial MBL changes in the CA group were 0.33 ± 0.10 mm at 6 months after the functional loading and 0.41 ± 0.13 mm at 12 months after the functional loading, which were significantly higher than those of the PA group, with 0.12 ± 0.04 mm (*p* = 0.031) and 0.14 ± 0.04 mm (*p* = 0.014), respectively. The distal MBL changes in the CA group were 0.30 ± 0.11 mm at 1 month after the functional loading, 0.41 ± 0.12 mm at 3 months, 0.40 ± 0.11 mm at 6 months, and 0.52 ± 0.14 mm at 12 months, which were significantly higher than those in the PA group, with 0.04 ± 0.03 mm (*p* = 0.006), 0.13 ± 0.05 mm (*p* = 0.013), 0.13 ± 0.05 mm (*p* = 0.013), and 0.14 ± 0.05 mm (*p* = 0.002), respectively (Table 8).

When comparing the average of the surrounding implant crestal bone level, no significant difference was found between the PA group and the CA group, as shown in the table. No significant difference was noted in the radiographs of the PA group and CA group. However, when comparing the MBL changes, the average mesiodistal change was found to be 0.20 ± 0.08 mm for the CA group, which was significantly higher than the 0.04 ± 0.03 mm in the PA group (*p* = 0.037), at 1 month after the functional loading. Over time, it was 0.46 ± 0.12 mm in the CA group, which was significantly higher than the 0.14 ± 0.04 mm in the PA group, at 12 months after the functional loading (Table 9).

### 3.6. Comparison of Groups by Implant Diameter

**Figure 6:**
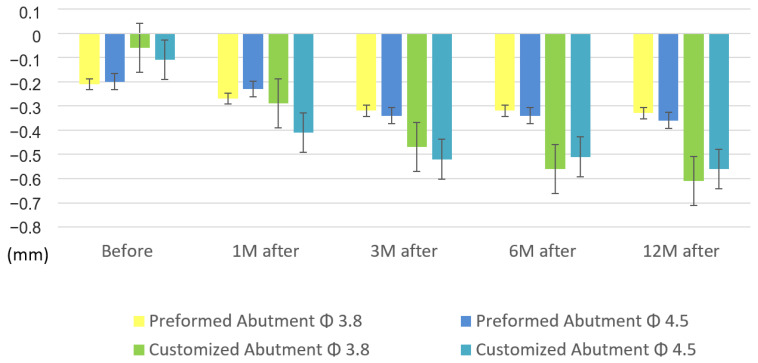
Comparison of groups by implant diameter of the PAs and CAs. CA, customized abutment; PA, preformed stock abutment.

The mesial and distal MBLs were similar between groups of diameters in the PA group, and no significant difference was found between the implants with diameters of 3.8 or 4.5 mm. However, in the comparison of the average MBLs, the MBL of the Φ 4.5 mm implants was 0.36 ± 0.08 mm at 12 months after the functional loading, which was slightly lower than that of the Φ 3.8 mm implants with 0.33 ± 0.09 mm. By contrast, the situation for the CA group was different; it was 0.61 ± 0.13 mm for the Φ 3.8 mm implants and 0.41 ± 0.16 mm for the Φ 4.5 mm implants. The MBL for the Φ 3.8 mm implants was slightly lower than for the Φ 4.5 mm implants (Figure 6).

In the CA group, the mesial and distal MBLs of the group with an implant diameter of 3.8 mm were lower than that of the group with an implant diameter of 4.5 mm, but no significant difference was found (Table 10).

### 3.7. Comparison of Groups by Implant Length

The results of the current analysis did not detect a significant difference in the MBL between the CA group and the PA group (Table 11), which meant that the bone level was not related to the implant length.

## 4. Discussion

During the 1-year study period, the PA group had a lower marginal bone loss (0.14 ± 0.04 mm) than the CA group (0.46 ± 0.12 mm) (*p* = 0.001). The CAD/CAM CAs revealed greater resorption on average, leading to a vertical bone level of 0.52 ± 0.41 mm at the end of the 1-year observation period, which was deeper than the vertical level in the PA group of 0.34 ± 0.06 mm.

Overall, the average MBL in the PA group was 0.34 ± 0.06 mm, which was similar to the value of 0.29 ± 0.85 mm in the relevant literature [14]. This demonstrated that the data collection in the present study was reliable and met the criteria for implant success determined by Albrektsson and Zarb.

Statistically, the distal marginal bone loss was higher than the mesial marginal bone loss in both the PA and CA groups. Previously, Emre Mucum et al. also mentioned that distal marginal bone loss is greater than mesial marginal bone loss [27], which may be because the posterior area is usually under higher loading due to occlusal forces than in the anterior area. These forces can also be higher on the distal side than on the mesial side. In contrast, it may be more difficult to clean the distal side well, and the maintenance of oral hygiene is more likely to be restrained.

In this study, the MBLs in the PA and CA groups measured on radiographs were comparable, which represented no significant difference in clinical outcomes. Nevertheless, the occlusal force affects the dental implants and the surrounding bone. According to bone physiology theory, the bone that carries the occlusal force adapts to the strength of the load through bone remodeling [28,29]. The bone level in each period was different, indicating the existence of the phenomenon of bone remodeling, and it starts to occur 1 month after functional loading. Moreover, the CA group had significantly more bone loss than the PA group, but the trend of bone loss itself was moderate, and no rapid or massive loss was found for any period. In contrast, the amount of mesial and distal marginal bone losses in the PA group was minimal, and no significant difference was noted. Studies showed that if the bone around the implants went through reabsorption because of implant overloading, implant failure may occur [30,31]. In each period in the CA group, the distal marginal bone loss was greater than the mesial marginal bone loss, which may have been related to whether the connection maintained the axial symmetry during the CA fabrication, whether the insertion path was deformed, or whether the mechanical structure was changed, leading to the micro-movement and pumping effects becoming concentrated at the distal side.

In a statistical analysis published by David French in 2019, an inverse relationship was observed between the implant diameter and bone loss; each 1 mm increase in implant diameter led to an approximately 0.11 mm reduction in bone loss [32]. In the CA group, the 4.5 mm diameter group with a 0.41 ± 0.16 mm bone level was better than the 3.8 mm diameter group with 0.61 ± 0.13 mm. By contrast, the results were the opposite in the PA group. No significant difference was noted between the implants with diameters of either 3.8 or 4.5 mm. In addition to the implant diameter, the maintenance of oral hygiene must be considered.

In the last decade, standard-length implants were shown to have a 98% success rate; the use of implants longer than 13 mm may cause slightly higher resorption due to poor bone quality [33]. In this study, standard lengths of 9.5 mm and 11 mm were used, and no significant bone resorption was found in either the PA group or the CA group.

In the present study, the limitations included the retrospective study design, the sample population, the use of two-dimensional (2D) digital radiographs, tracking only the period before the functional loading to after the functional loading, and the lack of intraoral occlusion records. The use of 2D radiographs was limited to the measurement of the mesiodistal MBL; thus, the buccolingual bone level and crestal bone volume around the dental implants could not be measured. In previous studies, the change in the buccolingual MBL and bone volume around implants could be examined using cone-beam computed tomography [34,35]. The crestal marginal bone resorption around dental implants is also associated with the surgical protocol used [36]. Another drawback was the lack of intraoral occlusal records to confirm the existence of overloading, which was reported as a potential threat to implant failure [30]. Thus, data integrity would be improved by increasing the sample size and number of observation items.

## 5. Conclusions

Within the limitations of this study, no clinically significant difference was noted in the MBL between the PAs and CAs after 1 year. Nevertheless, the change in the bone level of implants with the CAs was slightly higher than in the implants with the PAs.

## Figures and Tables

**Table 1:** Characteristics of the study participants.

Parameter		Preformed Abutments (*n* = 35)			Customized Abutments (*n* = 22)		
Age		53					
Patient							
			33			17	
Gender							
	Female	16		48.50%	11		64.70%
	Male	17		51.50%	6		35.30%

**Table 2:** Characteristics of the implants.

Parameter		Preformed Abutments (*n* = 35)		Customized Abutments (*n* = 22)	
Location					
	Upper jaw	17	48.60%	18	81.80%
	Lower jaw	18	51.40%	4	18.20%
Diameter					
	3.8 mm	17	48.60%	11	50.00%
	4.5 mm	18	51.40%	11	50.00%
Length					
	9.5 mm	10	28.60%	17	77.30%
	11 mm	25	71.40%	5	22.70%

**Table 3:** Mesial and distal bone levels in each period. SEM, standard error of measurement. (* *p* < 0.05, by two-tailed *t*-test.).

Type of Abutments	Time(Relative to Loading)	Mean ± SEM (mm)		*p* Value
		Mesial bone level	Distal bone level	
Preformed abutments				
	Before loading	0.10 ± 0.03	0.31 ± 0.07	0.012 *
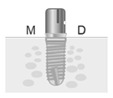	1 month after	0.15 ± 0.05	0.35 ± 0.07	0.029 *
	3 months after	0.22 ± 0.06	0.44 ± 0.08	0.027 *
	6 months after	0.22 ± 0.06	0.44 ± 0.08	0.024 *
	12 months after	0.24 ± 0.06	0.45 ± 0.08	0.041 *
Customized abutments				
	Before loading	0.10 ± 0.07	0.11 ± 0.05	0.917
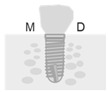	1 month after	0.20 ± 0.09	0.41 ± 0.11	0.134
	3 months after	0.34 ± 0.10	0.52 ± 0.12	0.267
	6 months after	0.42 ± 0.10	0.51 ± 0.11	0.547
	12 months after	0.48 ± 0.12	0.56 ± 0.12	0.641

**Table 4:** Mesial and distal marginal bone levels between the PAs and CAs. CA, customized abutment; PA, preformed stock abutment; SEM, standard error of measurement.

Location of Bone	Time(Relative to Loading)	Mean ± SEM (mm)		*p* Value
		Preformed	Customized	
Mesial bone level				
	Before loading	0.10 ± 0.03	0.10 ± 0.07	0.967
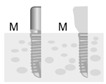	1 month after	0.15 ± 0.05	0.20 ± 0.09	0.629
	3 months after	0.22 ± 0.06	0.34 ± 0.10	0.279
	6 months after	0.22 ± 0.06	0.42 ± 0.10	0.064
	12 months after	0.24 ± 0.06	0.48 ± 0.12	0.052
Distal bone level				
	Before loading	0.31 ± 0.07	0.11 ± 0.05	0.051
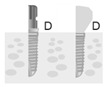	1 month after	0.35 ± 0.07	0.41 ± 0.11	0.619
	3 months after	0.44 ± 0.08	0.52 ± 0.12	0.536
	6 months after	0.44 ± 0.08	0.51 ± 0.11	0.588
	12 months after	0.45 ± 0.08	0.56 ± 0.12	0.428

**Table 5:** Mesial and distal marginal bone levels in the PAs. PA, preformed stock abutment; SEM, standard error of measurement.

Type of Abutments	Location of Bone	Time(Relative to Loading)	Mean ± SEM (mm)	*p* Value			
				Before loading	1M	3M	6M
Preformed abutment	Mesial bone level						
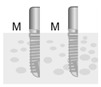		Before loading	0.10 ± 0.03				
		1 month after	0.15 ± 0.05	0.400			
		3 months after	0.22 ± 0.06	0.084	0.361		
		6 months after	0.22 ± 0.06	0.084	0.361	1.000	
		12 months after	0.24 ± 0.06	0.055	0.257	0.845	0.873
Preformed abutment	Distal bone level						
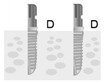		Before loading	0.31 ± 0.07				
		1 month after	0.35 ± 0.07	0.719			
		3 months after	0.44 ± 0.08	0.227	0.393		
		6 months after	0.44 ± 0.08	0.208	0.365	0.958	
		12 months after	0.45 ± 0.08	0.194	0.343	0.918	0.959

**Table 6:** Mesial and distal marginal bone levels in the CAs. CA, customized abutment; SEM, standard error of measurement.

Type of Abutments	Location of Bone	Time(Relative to Loading)	Mean ± SEM (mm)	*p* Value			
				Before loading	1M	3M	6M
Customized abutment	Mesial bone level						
		Before loading	0.10 ± 0.07				
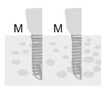		1 month after	0.20 ± 0.09	0.395			
		3 months after	0.34 ± 0.10	0.062	0.292		
		6 months after	0.42 ± 0.10	0.011	0.093	0.572	
		12 months after	0.48 ± 0.12	0.006	0.054	0.381	0.708
Customized abutment	Distal bone level						
		Before loading	0.11 ± 0.05				
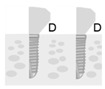		1 month after	0.41 ± 0.11	0.018			
		3 months after	0.52 ± 0.12	0.004	0.494		
		6 months after	0.51 ± 0.11	0.002	0.501	0.960	
		12 months after	0.56 ± 0.12	<0.001	0.358	0.824	0.771

**Table 7:** Change in the mesial and distal marginal bone levels in the PAs and CAs. CA, customized abutment; PA, preformed stock abutment; SEM, standard error of measurement.

Type of Abutments	Time(Relative to Loading)	Mean ± SEM (mm)		*p* Value
		Change in mesial	Change in distal	
Preformed abutments				
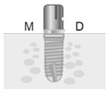	1 month after	0.05 ± 0.03	0.04 ± 0.03	0.792
	3 months after	0.12 ± 0.04	0.13 ± 0.05	0.850
	6 months after	0.12 ± 0.04	0.13 ± 0.05	0.781
	12 months after	0.14 ± 0.04	0.14 ± 0.05	0.964
Customized abutments				
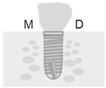	1 month after	0.10 ± 0.07	0.30 ± 0.11	0.119
	3 months after	0.24 ± 0.08	0.41 ± 0.12	0.139
	6 months after	0.33 ± 0.10	0.40 ± 0.11	0.626
	12 months after	0.41 ± 0.13	0.52 ± 0.14	0.575

**Table 8:** Comparison of the changes in the marginal bone levels between the PAs and CAs. CA, customized abutment; PA, preformed stock abutment; SEM, standard error of measurement. (* *p* < 0.05, ** *p* < 0.01, by two-tailed *t*-test.)

Location of Bone	Time(Relative to Loading)	Mean ± SEM (mm)		*p* Value
		Preformed	Customized	
Change in mesial bone				
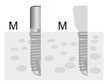	1 month after	0.05 ± 0.03	0.10 ± 0.07	0.507
	3 months after	0.12 ± 0.04	0.24 ± 0.08	0.132
	6 months after	0.12 ± 0.04	0.33 ± 0.10	0.031 *
	12 months after	0.14 ± 0.04	0.41 ± 0.13	0.014 *
Change in distal bone				
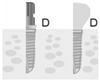	1 month after	0.04 ± 0.03	0.30 ± 0.11	0.006 **
	3 months after	0.13 ± 0.05	0.41 ± 0.12	0.013 *
	6 months after	0.13 ± 0.05	0.40 ± 0.11	0.014 *
	12 months after	0.14 ± 0.05	0.52 ± 0.14	0.002 **

**Table 9:** Comparison of the average changes in the surrounding implant crestal bone level between the PAs and CAs. CA, customized abutment; PA, preformed stock abutment; SEM, standard error of measurement (* *p* < 0.05, ** *p* < 0.01, by two-tailed *t*-test.).

Parameters	Time(relative to loading)	Mean ± SEM (mm)		*p* Value
		Preformed	Customized	
Marginal bone level				
	Before loading	0.21 ± 0.04	0.10 ± 0.04	0.123
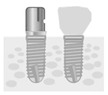	1 month after	0.25 ± 0.05	0.30 ± 0.09	0.566
	3 months after	0.33 ± 0.06	0.43 ± 0.11	0.354
	6 months after	0.33 ± 0.06	0.47 ± 0.10	0.193
	12 months after	0.34 ± 0.06	0.52 ± 0.10	0.111
Change in bone				
	1 month after	0.04 ± 0.03	0.20 ± 0.08	0.037 *
	3 months after	0.12 ± 0.04	0.33 ± 0.09	0.020 *
	6 months after	0.13 ± 0.04	0.36 ± 0.10	0.013 *
	12 months after	0.14 ± 0.04	0.46 ± 0.12	0.001 **

**Table 10:** Comparison of the mesiodistal marginal bone levels of the groups by the implant diameter of the CAs. CA, customized abutment; SEM, standard error of measurement.

Type of Abutments	Time(Relative to Loading)	Mean ± SEM (mm)		*p* Value
Customized abutments		Φ3.8 mm	Φ4.5 mm	
Mesial bone level	Before loading	0.09 ± 0.09	0.11 ± 0.11	0.899
	1 month after	0.25 ± 0.14	0.14 ± 0.11	0.510
	3 months after	0.42 ± 0.15	0.26 ± 0.15	0.475
	6 months after	0.54 ± 0.14	0.30 ± 0.14	0.251
	12 months after	0.57 ± 0.15	0.37 ± 0.20	0.430
Distal bone level				
	Before loading	0.04 ± 0.04	0.18 ± 0.10	0.169
	1 month after	0.33 ± 0.15	0.49 ± 0.16	0.468
	3 months after	0.53 ± 0.16	0.52 ± 0.20	0.972
	6 months after	0.58 ± 0.15	0.44 ± 0.16	0.532
	12 months after	0.64 ± 0.17	0.46 ± 0.17	0.462

**Table 11:** Comparison of groups by implant length. SEM, standard error of measurement.

Type of Abutments	Time(Relative to Loading)	Mean ± SEM (mm)		*p* Value
Length		9.5 mm	11 mm	
Customized abutments	Before loading	0.07 ± 0.04	0.22 ± 0.10	0.122
	1 month after	0.30 ± 0.11	0.32 ± 0.11	0.916
	3 months after	0.40 ± 0.13	0.54 ± 0.17	0.600
	6 months after	0.44 ± 0.11	0.60 ± 0.18	0.532
	12 months after	0.46 ± 0.11	0.78 ± 0.20	0.236
Preformed abutments				
	Before loading	0.29 ± 0.08	0.17 ± 0.05	0.235
	1 month after	0.29 ± 0.08	0.23 ± 0.06	0.604
	3 months after	0.42 ± 0.10	0.29 ± 0.07	0.331
	6 months after	0.42 ± 0.10	0.30 ± 0.07	0.350
	12 months after	0.44 ± 0.11	0.31 ± 0.07	0.301

## Data Availability

Not applicable.
